# Supplementary material for: HIVprotI: an integrated web based platform for prediction and design of HIV proteins inhibitors
Source: J Cheminform. 2018 Mar 9;10:12. doi: 10.1186/s13321-018-0266-y (PMC5845081; doi:10.1186/s13321-018-0266-y)
Supplement: Supplementary file 2 — Additional file 2. Source code of HIVProtI web server. [file 13321_2018_266_MOESM2_ESM.zip › HIVprotI_Source-code/hivprotI/weka-3-6-6/doc/weka/core/neighboursearch/PerformanceStats.html]

PerformanceStats


---


|  |  |  |
| --- | --- | --- |
| **Overview** \| **Package** \| **Class** \| **Tree** \| **Deprecated** \| **Index** \| **Help** \| **Weka's home** | |  |
| **PREV CLASS**   **NEXT CLASS** | **FRAMES**    **NO FRAMES**     **All Classes** |  |
| SUMMARY: NESTED \| FIELD \| CONSTR \| METHOD | DETAIL: FIELD \| CONSTR \| METHOD |  |


---


## weka.core.neighboursearch Class PerformanceStats

```
java.lang.Object
  weka.core.neighboursearch.PerformanceStats
```

**All Implemented Interfaces:**: java.io.Serializable, AdditionalMeasureProducer, RevisionHandler

**Direct Known Subclasses:**: TreePerformanceStats

---

``` public class PerformanceStats extends java.lang.Object implements AdditionalMeasureProducer, java.io.Serializable, RevisionHandler ```

The class that measures the performance of a nearest
neighbour search (NNS) algorithm.

**Version:**
:   $Revision: 1.2 $

**Author:**
:   Ashraf M. Kibriya (amk14[at-the-rate]cs[dot]waikato[dot]ac[dot]nz)

**See Also:**: Serialized Form

---

| **Field Summary** | |
| --- | --- |
| `double` | `m_CoordCount`             The number of coordinates looked at for the current/last query. |
| `double` | `m_MaxC`             The min and max coordinates(attributes) looked at per query. |
| `double` | `m_MaxP`             The min and max data points looked for a query by the NNS algorithm. |
| `double` | `m_MinC`             The min and max coordinates(attributes) looked at per query. |
| `double` | `m_MinP`             The min and max data points looked for a query by the NNS algorithm. |
| `double` | `m_PointCount`             The number of data points looked at for the current/last query. |
| `double` | `m_SumC`             The sum of coordinates/attributes looked at for all the queries. |
| `double` | `m_SumP`             The sum of data points looked at for all the queries. |
| `double` | `m_SumSqC`             The squared sum of coordinates/attributes looked at for all the queries. |
| `double` | `m_SumSqP`             The squared sum of data points looked at for all the queries. |


**Constructor Summary**

`PerformanceStats()`
  
          default constructor.


| **Method Summary** | |
| --- | --- |
| `java.util.Enumeration` | `enumerateMeasures()`             Returns an enumeration of the additional measure names. |
| `double` | `getMaxCoordsPerPoint()`             Returns the maximum of coords per point. |
| `double` | `getMaxPointsVisited()`             Returns the maximum of points visited. |
| `double` | `getMeanCoordsPerPoint()`             Returns the mean of coords per point. |
| `double` | `getMeanPointsVisited()`             Returns the mean of points visited. |
| `double` | `getMeasure(java.lang.String additionalMeasureName)`             Returns the value of the named measure. |
| `double` | `getMinCoordsPerPoint()`             Returns the minimum of coords per point. |
| `double` | `getMinPointsVisited()`             Returns the minimum of points visited. |
| `int` | `getNumQueries()`             Returns the number of queries. |
| `java.lang.String` | `getRevision()`             Returns the revision string. |
| `java.lang.String` | `getStats()`             Returns a string representation of the statistics. |
| `double` | `getStdDevCoordsPerPoint()`             Returns the standard deviation of coords per point. |
| `double` | `getStdDevPointsVisited()`             Returns the standard deviation of points visited. |
| `double` | `getTotalCoordsPerPoint()`             Returns the total sum of coords per point. |
| `double` | `getTotalPointsVisited()`             Returns the total number of points visited. |
| `void` | `incrCoordCount()`             Increments the coordinate count (number of coordinates/attributes looked at). |
| `void` | `incrPointCount()`             Increments the point count (number of datapoints looked at). |
| `void` | `reset()`             Resets all internal fields/counters. |
| `void` | `searchFinish()`             Signals end of the nearest neighbour search. |
| `void` | `searchStart()`             Signals start of the nearest neighbour search. |
| `void` | `updatePointCount(int n)`             adds the given number to the point count. |

**Methods inherited from class java.lang.Object**

`equals, getClass, hashCode, notify, notifyAll, toString, wait, wait, wait`

**Field Detail**

### m\_MinP

```
public double m_MinP
```

:   The min and max data points looked for a query by
    the NNS algorithm.

---


### m\_MaxP

```
public double m_MaxP
```

:   The min and max data points looked for a query by
    the NNS algorithm.

---


### m\_SumP

```
public double m_SumP
```

:   The sum of data points looked
    at for all the queries.

---


### m\_SumSqP

```
public double m_SumSqP
```

:   The squared sum of data points looked
    at for all the queries.

---


### m\_PointCount

```
public double m_PointCount
```

:   The number of data points looked at
    for the current/last query.

---


### m\_MinC

```
public double m_MinC
```

:   The min and max coordinates(attributes) looked
    at per query.

---


### m\_MaxC

```
public double m_MaxC
```

:   The min and max coordinates(attributes) looked
    at per query.

---


### m\_SumC

```
public double m_SumC
```

:   The sum of coordinates/attributes looked at
    for all the queries.

---


### m\_SumSqC

```
public double m_SumSqC
```

:   The squared sum of coordinates/attributes looked at
    for all the queries.

---


### m\_CoordCount

```
public double m_CoordCount
```

:   The number of coordinates looked at for
    the current/last query.


**Constructor Detail**

### PerformanceStats

```
public PerformanceStats()
```

:   default constructor.


**Method Detail**

### reset

```
public void reset()
```

:   Resets all internal fields/counters.

---


### searchStart

```
public void searchStart()
```

:   Signals start of the nearest neighbour search.
    Initializes the stats object.

---


### searchFinish

```
public void searchFinish()
```

:   Signals end of the nearest neighbour search.
    Calculates the statistics for the search.

---


### incrPointCount

```
public void incrPointCount()
```

:   Increments the point count
    (number of datapoints looked at).

---


### incrCoordCount

```
public void incrCoordCount()
```

:   Increments the coordinate count
    (number of coordinates/attributes
    looked at).

---


### updatePointCount

```
public void updatePointCount(int n)
```

:   adds the given number to the point count.

    :   **Parameters:**: `n` - The number to add to the point count.

---


### getNumQueries

```
public int getNumQueries()
```

:   Returns the number of queries.

    :   **Returns:**: The number of queries.

---


### getTotalPointsVisited

```
public double getTotalPointsVisited()
```

:   Returns the total number of points visited.

    :   **Returns:**: The total number.

---


### getMeanPointsVisited

```
public double getMeanPointsVisited()
```

:   Returns the mean of points visited.

    :   **Returns:**: The mean points visited.

---


### getStdDevPointsVisited

```
public double getStdDevPointsVisited()
```

:   Returns the standard deviation of points visited.

    :   **Returns:**: The standard deviation.

---


### getMinPointsVisited

```
public double getMinPointsVisited()
```

:   Returns the minimum of points visited.

    :   **Returns:**: The minimum.

---


### getMaxPointsVisited

```
public double getMaxPointsVisited()
```

:   Returns the maximum of points visited.

    :   **Returns:**: The maximum.

---


### getTotalCoordsPerPoint

```
public double getTotalCoordsPerPoint()
```

:   Returns the total sum of coords per point.

    :   **Returns:**: The total per point.

---


### getMeanCoordsPerPoint

```
public double getMeanCoordsPerPoint()
```

:   Returns the mean of coords per point.

    :   **Returns:**: The mean.

---


### getStdDevCoordsPerPoint

```
public double getStdDevCoordsPerPoint()
```

:   Returns the standard deviation of coords per point.

    :   **Returns:**: The standard deviation.

---


### getMinCoordsPerPoint

```
public double getMinCoordsPerPoint()
```

:   Returns the minimum of coords per point.

    :   **Returns:**: The minimum.

---


### getMaxCoordsPerPoint

```
public double getMaxCoordsPerPoint()
```

:   Returns the maximum of coords per point.

    :   **Returns:**: The maximum.

---


### enumerateMeasures

```
public java.util.Enumeration enumerateMeasures()
```

:   Returns an enumeration of the additional measure names.

    :   **Specified by:**: `enumerateMeasures` in interface `AdditionalMeasureProducer`
    :   **Returns:**: An enumeration of the measure names.

---


### getMeasure

```
public double getMeasure(java.lang.String additionalMeasureName)
                  throws java.lang.IllegalArgumentException
```

:   Returns the value of the named measure.

    :   **Specified by:**: `getMeasure` in interface `AdditionalMeasureProducer`
    :   **Parameters:**: `additionalMeasureName` - The name of the measure to query for its value. **Returns:**: The value of the named measure. **Throws:**: `java.lang.IllegalArgumentException` - If the named measure is not supported.

---


### getStats

```
public java.lang.String getStats()
```

:   Returns a string representation of the statistics.

    :   **Returns:**: The statistics as string.

---


### getRevision

```
public java.lang.String getRevision()
```

:   Returns the revision string.

    :   **Specified by:**: `getRevision` in interface `RevisionHandler`
    :   **Returns:**: the revision


---


|  |  |  |
| --- | --- | --- |
| **Overview** \| **Package** \| **Class** \| **Tree** \| **Deprecated** \| **Index** \| **Help** \| **Weka's home** | |  |
| **PREV CLASS**   **NEXT CLASS** | **FRAMES**    **NO FRAMES**     **All Classes** |  |
| SUMMARY: NESTED \| FIELD \| CONSTR \| METHOD | DETAIL: FIELD \| CONSTR \| METHOD |  |


---
